# Fluconazole resistant pathogenic yeasts isolated from plastic debris on recreational public beaches in West and East Africa

**DOI:** 10.1007/s11756-025-01981-w

**Published:** 2025-06-21

**Authors:** Ayorinde B. Akinbobola, Dativa Joseph Shilla, Daniel A. Shilla, Richard S. Quilliam

**Affiliations:** 1 Biological and Environmental Sciences, Faculty of Natural Sciences, University of Stirling, Stirling, FK9 4LA UK; 2 Department of Microbiology, Adekunle Ajasin University, Akungba-Akoko, Nigeria; 3 Department of Chemistry, Dar es-Salaam University College of Education (DUCE-UDSM), Dar es Salaam, Tanzania; 4 School of Aquatic Sciences and Fisheries Technology, University of Dar es Salaam, Dar es Salaam, Tanzania

**Keywords:** *Candida*, Fungal pathogens, Nigeria, Plastisphere, Tanzania, WHO fungal priority pathogens list

## Abstract

Plastic pollution in the environment becomes rapidly colonised by microbial communities, which often contain human bacterial pathogens. However, there is a lack of information about the interaction of fungal pathogens with plastic debris, particularly in marine environments. This study screened common plastic wastes collected from a range of recreational public and tourist beaches in Nigeria and Tanzania for colonisation by human pathogenic yeasts. Isolates were identified on selective media with confirmation by ITS sequencing. All beaches and all plastic polymer types were colonised by at least one species of human pathogenic yeast, with *Candida tropicalis* being the most frequently isolated species across both countries. Importantly, most of these pathogenic yeast isolates showed some level of resistance to fluconazole, which in Africa is the most commonly prescribed anti-fungal drug. Therefore, due to the high potential for human skin exposure at beach environments, plastic debris could pose a significant public health risk.

## Introduction

The projected increase in the global production of plastic will inevitably lead to greater volumes of plastic waste being released into the environment, particularly in low- and middle-income countries (LMICs), where there has been a lack of investment in waste management that is often accompanied by poor regulation of plastic use and disposal (Mphasa et al. 2025; Yan et al. 2024). Once in the environment, microbial biofilm rapidly colonises plastic debris to form plastic-associated microbial communities known as the ‘plastisphere’ (Zettler et al. 2013). Such biofilm communities can also support potentially pathogenic viruses and bacteria and act as a vector to facilitate their dissemination in the environment (Metcalf et al. 2022; Moresco et al. 2022). Therefore, plastic wastes may be a major factor facilitating the environmental survival of pathogens, antimicrobial resistance (AMR) genes, and even the emergence of novel zoonotic diseases (Ormsby et al. 2024).

In contrast to pathogenic bacteria, the incidence of human pathogenic fungi in the plastisphere is less well-studied (Ormsby et al. 2023; Gkoutselis et al. 2021). Human pathogenic yeasts, such as species of *Candida*, are well known for their production of strong biofilms on the surfaces of plastic in clinical settings (de Barros et al. 2020), and *Candida* species can also withstand diverse environmental conditions and persist in a range of different environmental matrices (Opulente et al. 2019). Globally, fungal pathogens are responsible for 1.7 million deaths per year, and approximately 150 million severe cases of infection (Kainz et al. 2020). Species of *Candida* are responsible for an estimated 700,000 annual cases of invasive candidiasis (Bongomi et al. 2017), in addition to the burden of less severe cases of cutaneous and mucosal infections, such as thrush. Although *Candida albicans* is the most common aetiological agent of candidiasis, there has been an increase in cases of candidiasis caused by non-*albicans* species (Sharma and Chakrabarti 2023).

Globally, infections due to pathogenic *Candida* species are increasing, due in part to the simultaneous emergence of more virulent strains with increasing anti-fungal drug resistance (Arastehfar et al. 2020; Siscar-Lewin et al. 2022). Concurrently, the human population that are susceptible to opportunistic pathogenic yeast infections has also increased, e.g., due to an increasingly aging population and underlying conditions such as cancer, autoimmune disease, and chronic infections (Koehler et al. 2019). The critical importance of emerging drug-resistant and more virulent strains of *Candida* is reflected in the WHO Fungal Pathogen Priority List (which includes, *C. auris*, *C. albicans*, *C. glabrata*, *C. parapsilosis*, and *C. tropicalis*) that was recently compiled to guide research and public health action (WHO 2022). Importantly, however, we know almost nothing about the environmental survival and transfer pathways of pathogenic species of *Candida* in natural environments.

It has recently been estimated that in Africa and Asia, there is a disproportionately high accumulation of plastic waste compared to other continents, with Nigeria and Tanzania ranked 6th and 7th in the world and 1 st and 2nd in Africa respectively for annual generation of mismanaged plastic waste, most of which will end up in the ocean (Lebreton and Andrady 2019). Dissemination of plastic waste through the landscape is facilitated by their lightweight and buoyant properties, which can also increase the likelihood of human exposure to plastics contaminated with potential pathogens. The aim of this study, therefore, was to quantify the prevalence of pathogenic species of *Candida* on plastic debris at frequently used public and tourist beaches in Nigeria and Tanzania by screening commonly found items of plastic pollution. In addition, we aimed to determine the level of resistance to fluconazole (one of the commonly used antifungal drugs for the treatment of fungal infections) in these pathogenic yeasts isolated at the beach.

## Methods and materials

### Sample collection and processing

In Nigeria, five beaches in Lagos (which is the most populated city in Nigeria with an estimated population size of 15 million people), and one coastal site in Ondo state Nigeria, were selected because they are frequently used for recreational purposes. In Tanzania, a tourist beach in Stone Town on the island of Zanzibar in the Indian Ocean, and a local public beach in Dar es Salaam were selected. Zanzibar is a popular destination for tourists worldwide (with over 600,000 visitors annually), and Dar es Salaam is the capital city of Tanzania and its largest city with an estimated population of over 8 million people. No authority or permits were required for access to these public beaches, and ethical approval for this project was authorised by the General University Ethics Panel (Ref. GUEP 6748). Using gloves, plastic debris were collected from the intertidal zone of each beach and transported back to the laboratory in sample bags. All plastic items were subsequently sorted by polymer type, i.e., polyethene terephthalate (PET), high-density polyethene (HDPE), polyvinyl chloride (PVC), low-density polyethene (LDPE), polypropylene (PP), or polystyrene (PS).

### Isolation of pathogenic species of *Candida*

Plastic items of the same polymer from each site were made up into a composite sample (depending on volume of plastic wastes at each beach, sometimes there were several composite samples of the same polymer from each site) and added to flasks containing a pre-enrichment broth of yeast extract peptone dextrose (YPD) (Sigma-Aldrich, USA) supplemented with 50 mg/L of chloramphenicol and gentamycin (Sigma-Aldrich, USA), and incubated at 37 °C. After 48 h, the broth from each flask was serially diluted and 0.1 mL of three dilutions (0, 10^–3^ and 10^–5^) was spread on Petri dishes containing Candida Plus CHROMagar (CHROMagar™, France), and incubated aerobically at 37 °C. After 48 h of incubation, colonies showing morphologies characteristic of human pathogenic *Candida* species on Candida Plus CHROMagar (Nadeem et al. 2010) were selected and sub-cultured onto fresh Candida Plus CHROMagar to obtain pure cultures.

### Identification of yeast isolates

Presumptive identification of isolates was based on their cultural morphology on Candida Plus CHROMagar, followed by confirmation based on ITS sequencing. Inoculum from pure cultures was preserved in DNA/RNA Shield (ZymoResearh, USA) for storage, and subsequent DNA extraction carried out using the Quick-DNA™ MiniPrep (Zymo Research, USA). The ITS region was amplified using the ITS primers described in Trost et al. (2004) (Forward primer: 5’-GTCAAACTTGGTCATTTA-3’; Reverse primer: 5’-TTCTTTTCCTCCGCTTATTG-3’), in a 50 µL PCR reaction comprised of: 25 µL master mix (Qiagen, Germany), 2 µL of forward and reverse primers (10 µmol/L), 10 µL of DNA template and 11 µL of PCR grade water. The PCR conditions consisted of a single initial denaturation at 94 °C for 3 min followed by 34 cycles of 30 s at 94 °C, 30 s at 50 °C and 60 s at 72 °C followed by a final extension at 72 °C for 10 min. The PCR products were purified with the QIAquick PCR Purification kit (Qiagen, Germany), and run down a 2% agarose gel stained with GelRed® (Biotium, USA) and visualised under UV with a 1 Kb Plus DNA ladder (Invitrogen). The amplified ITS region of the purified PCR products was sequenced using Applied Biosystems 3730 DNA analysers (DNA Sequencing and Services, University of Dundee, UK). Matches to sequences of all isolates were made using the NCBI Basic Local Alignment Search Tool (BLAST). Sequences have been deposited in Genbank under accession numbers PP808732 to PP808759 for isolates from Nigerian beaches and PQ677004 to PQ677035 for isolates from Tanzanian beaches.

### Susceptibility of yeast isolates to fluconazole

Inoculum used for the susceptibility test was prepared from cells recovered by centrifugation at 4000 rpm for 8 min from overnight cultures grown in YPD; cells were washed and resuspended in PBS to obtain a final concentration of 10^5^ CFU/mL. Susceptibility to the anti-fungal drug fluconazole was quantified using the agar dilution method as described by Gomaa et al. (2024). Fluconazole was added (to give final concentrations of 0, 4, 8, 16, 32 and 64 mg/L) to freshly prepared Sabouraud Glucose Agar (SGA; Sigma-Aldrich, USA) at approx. 45–50 °C. The fluconazole-supplemented agar was poured into Petri dishes, which were allowed to dry before inoculation with 20 µL of inoculum prepared from an overnight culture of each *Candida* isolate. Plates were incubated at 37 °C and examined for growth after 24 h.

## Results and discussion

Beaches in both Nigeria and Tanzania are frequently used for recreational and religious activities, yet debris of all five plastic polymers collected from all beaches sampled in this study were colonised by at least one species of yeast known to cause human infections. In total, 23 isolates of human pathogenic yeasts were recovered from plastic debris collected from Nigerian beaches (Table 1), and 38 isolates recovered from plastic wastes from the Tanzanian beaches (Table 2). More than 80% of all isolates were *C. tropicalis*, which is a common non-albicans etiological agent for candidiasis (Pfaller et al. 2010). Isolates of *Candida* species and other potential human pathogenic yeasts included *C. albicans*, *C. orthopsilosis*, *C. krusei* (now called *Pichia kudriavzevii*) and *Kodamaea ohmeri*, *Diutina rugosa* and *Moesziomyces aphidis*.

**Table 1:** Identity and fluconazole susceptibility of pathogenic yeasts isolated from plastic wastes on Nigerian beaches

Site	Polymer	Species	Accession no.	Fluconazole (mg/L)					
				0	4	8	16	32	64
N1	LDPE	*Candida tropicalis*	PP808732	+	+	NG	NG	NG	NG
N1	LDPE	*Candida tropicalis*	PP808733	+	NG	NG	NG	NG	NG
N1	LDPE	*Candida tropicalis*	PP808734	+	+	+	+	+	NG
N1	PET	*Candida tropicalis*	PP808735	+	+	+	NG	NG	NG
N1	HDPE	*Candida tropicalis*	PP808736	+	+	+	+	NG	NG
N2	LDPE	*Candida tropicalis*	PP808739	+	+	+	+	NG	NG
N2	LDPE	^*a*^*Pichia kudriavzevii*	PP808740	+	+	+	+	+	NG
N3	LDPE	*Candida orthopsilosis*	PP808741	+	NG	NG	NG	NG	NG
N3	LDPE	*Candida tropicalis*	PP808744	+	+	NG	NG	NG	NG
N3	LDPE	*Candida tropicalis*	PP808745	+	+	+	+	+	NG
N3	LDPE	*Candida tropicalis*	PP808746	+	+	+	NG	NG	NG
N3	PET	*Kodamaea ohmeri*	PP808747	+	+	+	NG	NG	NG
N3	PET	*Candida tropicalis*	PP808748	+	+	NG	NG	NG	NG
N3	HDPE	*Candida tropicalis*	PP808749	+	+	+	+	NG	NG
N3	HDPE	*Candida tropicalis*	PP808750	+	NG	NG	NG	NG	NG
N3	PS	*Candida tropicalis*	PP808751	+	+	+	NG	NG	NG
N3	PS	*Candida tropicalis*	PP808752	+	+	+	NG	NG	NG
N4	HDPE	*Candida tropicalis*	PP808754	+	NG	NG	NG	NG	NG
N5	LDPE	*Candida tropicalis*	PP808755	+	+	+	+	NG	NG
N5	LDPE	*Candida tropicalis*	PP808756	+	+	NG	NG	NG	NG
N6	PET	*Candida albicans*	PP808757	+	+	+	NG	NG	NG
N6	PP	*Candida tropicalis*	PP808758	+	NG	NG	NG	NG	NG
N6	HDPE	*Candida tropicalis*	PP808759	+	NG	NG	NG	NG	NG

^a^Formerly known as *Candida krusei*; NG no-growth

**Table 2:** Identity and fluconazole susceptibility of pathogenic yeasts isolated from plastic wastes on Tanzanian beaches

Site	Polymer	Species	Accession no	Fluconazole (mg/L)					
				0	4	8	16	32	64
T1	HDPE	*Candida tropicalis*	PQ677004	+	+	+	+	+	+
T1	HDPE	*Candida tropicalis*	PQ677005	+	+	NG	NG	NG	NG
T1	HDPE	^*a*^*Diutina rugosa*	PQ677006	+	+	+	+	NG	NG
T1	LDPE	^*b*^*Moesziomyces aphidis*	PQ677007	+	+	+	+	+	+
T1	PS	*Candida tropicalis*	PQ677008	+	+	+	+	NG	NG
T1	PS	*Candida tropicalis*	PQ677009	+	+	+	+	+	+
T2	HDPE	*Candida tropicalis*	PQ677014	+	+	+	+	+	NG
T2	HDPE	^*c*^*Pichia kudriavzevii*	PQ677015	+	+	+	+	+	+
T2	HDPE	*Candida tropicalis*	PQ677016	+	+	+	+	+	NG
T2	HDPE	*Candida tropicalis*	PQ677017	+	+	+	+	+	+
T2	LDPE	*Candida tropicalis*	PQ677018	+	+	+	+	+	NG
T2	LDPE	*Candida tropicalis*	PQ677019	+	+	+	+	+	NG
T2	LDPE	*Candida tropicalis*	PQ677020	+	+	+	+	+	+
T2	LDPE	*Candida tropicalis*	PQ677021	+	+	+	NG	NG	NG
T2	LDPE	*Candida tropicalis*	PQ677022	+	+	+	+	+	+
T2	LDPE	*Candida tropicalis*	PQ677023	+	+	+	+	NG	NG
T2	PP	*Candida tropicalis*	PQ677024	+	+	+	+	+	+
T3	PP	*Candida tropicalis*	PQ677025	+	+	+	+	+	+
T3	PP	*Candida tropicalis*	PQ677026	+	+	+	+	+	+
T3	PP	*Candida tropicalis*	PQ677027	+	+	+	+	+	+
T3	PP	*Candida tropicalis*	PQ677028	+	+	+	+	+	+
T3	PP	*Pichia kudriavzevii*	PQ677029	+	+	+	+	+	+
T3	PP	*Candida tropicalis*	PQ677030	+	+	+	+	+	+
T3	PP	*Candida tropicalis*	PQ677031	+	+	+	+	+	+
T3	PS	*Candida tropicalis*	PQ677032	+	+	+	+	+	NG
T3	PS	*Candida tropicalis*	PQ677033	+	+	+	+	+	NG
T3	PS	*Candida tropicalis*	PQ677034	+	+	+	+	+	+
T3	PS	*Candida tropicalis*	PQ677035	+	+	+	+	+	+

^a^Formerly known as *Candida rugosa;* ^b^Formerly known as *Pseudozyma aphidis*; ^c^Formerly known as *Candida krusei*; NGno-growth

*C. albicans* is the most commonly reported etiological agent of candidiasis globally including in sub-Saharan African countries like Nigeria (Okoye et al. 2022). *C. albicans*, which together with *C. tropicalis* and *P. kudriavzevii* are part of the five species responsible for most cases of global candidiasis, are all listed on the WHO Fungal Pathogen Priority List of fungal pathogens (WHO 2022). Although *P. kudriavzevii* is less common globally, its infections are complicated by innate resistance to fluconazole, the antifungal drug commonly used in the treatment of *Candida* infections (Douglass et al. 2018).

The frequency that *C*. *tropicalis* was recovered from plastic wastes at beaches in both West and East Africa is probably a combination of the capacity for *C. tropicalis* to adhere to surfaces and form strong biofilms, which confers protection against environmental stressors, together with their high level of tolerance to the conditions common at coastal environments and tropical climates (Zuza-Alves et al. 2019). *C. tropicalis* can tolerate high salt concentrations and survive in hypersaline environments (Lima et al. 2022) and are more commonly found in the tropics compared to other pathogenic *Candida* species. Consequently, *C. tropicalis* infections are also more prevalent in tropical regions compared to temperate regions (Bohner et al. 2021), and globally *C. tropicalis* is widely described as the second or third most common pathogenic yeast causing both superficial and systemic infections in humans (Zuza-Alves et al. 2019). The widespread colonisation of plastic debris collected from all beaches suggests that this common substrate could be playing an important role for the survival and subsequent epidemiology of *C. tropicalis* in tropical environments. Most of the *C. tropicalis* strains isolated from beaches in Nigeria showed some level of resistance to fluconazole at concentrations of 4 and 8 mg/L; however, the majority of isolates were unable to grow on SGA containing 16 mg/L of fluconazole (Table 1), and no isolates could grow on SGA containing 64 mg/L of fluconazole. Importantly, some strains *C. tropicalis* isolated from Tanzanian beaches could tolerate fluconazole at 64 mg/L and thus could pose a significant public health risk.

Emerging resistance to antifungal drugs is making *Candida* infections a serious global health concern. In comparison to antibiotics, only three classes of antifungal drugs (azoles, echinocandins, and polyenes) are available for treating fungal infections, and it has been hypothesised that the release of antifungal chemicals, such as azoles, into the environment (from both agricultural sources and pharmaceuticals in wastewater) are likely to result in the environmental enrichment of resistant strains of *Candida* (Fisher et al. 2018). Globally, azole antifungal drugs are vital for the treatment of fungal infections; however, resistance to common azoles like fluconazole is becoming more common (Arastehfar et al. 2020; Pathadka et al. 2022), but due to the cost, there is unequitable access to non-azole drugs in LMICs. Importantly, *P. kudriavzevii* (formally called *Candida krusei*) and some of the *C. tropicalis* isolates in this study exhibited resistance to fluconazole. *P. kudriavzevii* has an innate resistance to fluconazole (Douglass et al. 2018), but the isolation of *C. tropicalis* with reduced susceptibility to fluconazole in this study demonstrates the risk of plastic pollution disseminating resistant strains in the environment, particularly in areas such as recreational and public beaches where there is a high potential for contact with humans.

A single culture of both *Kodamaea ohmeri* and *C. orthopsilosis* were also isolated from beaches in Nigeria. *K. ohmeri*, which showed some resistance to fluconazole, has been reported as an emerging human pathogen capable of causing severe infections with high mortality rates worldwide (Silva et al. 2025; Zhou et al. 2021); although rarely reported in sub-Saharan African countries, *K. ohmeri* has been detected in Nigeria in individuals with superficial fungal infections (Joseph et al. 2022). *C. orthopsilosis* is commonly described as part of the *Candida parapsilosis* species complex and infections can be fatal in susceptible hosts and have been reported worldwide (Lockhart et al. 2008). Three strains of *Diutina rugosa* were isolated from Tanzanian beaches, and although *D. rugosa* infections in humans are rare, they have been reported to cause opportunistic infections in patients who have undergone invasive medical treatment (Paredes et al. 2012). Although there was evidence for resistance to fluconazole in the oslates of *D. rugosa* in this study, none of them could tolerate 32 mg/L of fluconazole. Finally, a single isolate of *Moesziomyces aphidi* with tolerance to fluconazole was also isolated from plastic debris at a beach in Tanzania. Although *Moesziomyces aphidi* is a known plant pathogen, there are reports that it can also cause rare infections in immunocompromised patients (de Carvalho Parahym et al. 2013; Mpakosi et al. 2022).

This study has demonstrated, for the first time, that plastic pollution at recreational coastal beaches in both West and East Africa can harbour important species of pathogenic *Candida*. The increasing influx of plastic wastes into the natural environment could therefore be facilitating the persistence and distribution of pathogenic microorganisms such as *Candida* (Baker et al. 2024; Metcalf et al. 2025) and amplifying the potential for human exposure to pathogens carrying resistance to anti-fungal drugs. Geographical variations in the common aetiological agents of candidiasis further suggest that environmental reservoirs play an important role in the epidemiology of *Candida* infections. This is exemplified by the recent emergence of the multi-drug resistant *Candida auris*, which may have originated in coastal environments (Casadevall et al. 2019; Akinbobola et al. 2023), and in experimental conditions has been shown to persist on plastics in seawater and beach sand for significant periods of time (Akinbobola et al. 2024). The urgency needed to increase our understanding of the implications of marine plastic debris as a vector for important human fungal pathogens is underlined by the recent WHO Fungal Pathogen Priority List, and here we provide the preliminary evidence that there is significant potential for human exposure to pathogenic species of *Candida* at polluted recreational beaches.

## Data Availability

Data will be made available on request.

## References

[CR1] Akinbobola AB, Kean R, Hanifi SMA, Quilliam RS (2023) Environmental reservoirs of the drug-resistant pathogenic yeast *Candida auris*. PLoS Pathog 19:e1011268. 10.1371/journal.ppat.1011268 37053164 10.1371/journal.ppat.1011268 PMC10101498

[CR2] Akinbobola A, Kean R, Quilliam RS (2024) Plastic pollution as a novel reservoir for the environmental survival of the drug resistant fungal pathogen *Candida auris*. Mar Pollut Bull 198:115841. 10.1016/j.marpolbul.2023.115841 38061145 10.1016/j.marpolbul.2023.115841

[CR3] Arastehfar A, Gabaldón T, Garcia-Rubio R, Jenks JD, Hoenigl M, Salzer HJ, Ilkit M, Lass-Flörl C, Perlin DS (2020) Drug-resistant fungi: an emerging challenge threatening our limited antifungal armamentarium. Antibiotics 9:877. 10.3390/antibiotics9120877 33302565 10.3390/antibiotics9120877 PMC7764418

[CR4] Baker T, Bester A, Sebolai O, Albertyn J, Pohl C (2024) Biofilms on urban aquatic plastic pollution as a reservoir for pathogenic yeasts. J Water Health 22(10):1826–1842. 10.2166/wh.2024.133

[CR5] Bohner F, Gacser A, Toth R (2021) Epidemiological attributes of *Candida* species in tropical regions. Cur Trop Med Rep 8:59–68. 10.1007/s40475-021-00226-5

[CR6] Bongomi F, Gago S, Oladele RO, Denning DW (2017) Global and multi-national prevalence of fungal diseases - estimate precision. J Fungi 3(4):57. 10.3390/jof3040057 10.3390/jof3040057 PMC5753159 29371573

[CR7] Casadevall A, Kontoyiannis DP, Robert V (2019) On the emergence of *Candida auris*: climate change, azoles, swamps, and birds. Mbio 10:1128. 10.1128/mbio.01397-19 10.1128/mBio.01397-19 PMC6650554 31337723

[CR8] de Barros PP, Rossoni RD, de Souza CM, Scorzoni L, Fenley JDC, Junqueira JC (2020) Candida biofilms: an update on developmental mechanisms and therapeutic challenges. Mycopathologia 185:415–424. 10.1007/s11046-020-00445-w 32277380 10.1007/s11046-020-00445-w

[CR9] de Carvalho Parahym AMR, da Silva CM, de Farias Domingos I, Gonçalves SS, de Melo Rodrigues M, de Morais VLL, Neves RP (2013) Pulmonary infection due to *Pseudozyma aphidis* in a patient with Burkitt lymphoma: first case report. Diagn Microbiol Infect Dis 75(1):104–106 23182077 10.1016/j.diagmicrobio.2012.09.010

[CR10] Douglass AP, Offei B, Braun-Galleani S, Coughlan AY, Martos AAR, Ortiz-Merino RA, Byrne KP, Wolfe KH (2018) Population genomics shows no distinction between pathogenic *Candida krusei* and environmental *Pichia kudriavzevii*: one species, four names. PLoS Pathog 14(7):e1007138. 10.1371/journal.ppat.1007138 30024981 10.1371/journal.ppat.1007138 PMC6053246

[CR11] Fisher MC, Hawkins NJ, Sanglard D, Gurr SJ (2018) Worldwide emergence of resistance to antifungal drugs challenges human health and food security. Science 360(6390):739–742. 10.1126/science.aap7999 29773744 10.1126/science.aap7999

[CR12] Gkoutselis G, Rohrbach S, Harjes J, Obst M, Brachmann A, Horn MA, Rambold G (2021) Microplastics accumulate fungal pathogens in terrestrial ecosystems. Sci Rep 11(1). 10.1038/s41598-021-92405-7 10.1038/s41598-021-92405-7 PMC8282651 34267241

[CR13] Gomaa SE, Abbas HA, Mohamed FA, Ali MAM, Ibrahim TM, Abdel Halim AS, Alghamdi MA, Mansour B, Chaudhary AA, Elkelish A (2024) The anti-staphylococcal fusidic acid as an efflux pump inhibitor combined with fluconazole against vaginal candidiasis in mouse model. BMC Microbiol 24:54. 10.1186/s12866-024-03181-z 38341568 10.1186/s12866-024-03181-z PMC10858509

[CR14] Joseph OV, Agbagwa OE, Frank-Peterside N (2022) The prevalence of fungal infections in six communities in Akwa Ibom State Nigeria. Afr J Health Sci 35(5):574–585. 10.61090/aksujacog.2024.003

[CR15] Kainz K, Bauer MA, Madeo F, Carmona-Gutierrez D (2020) Fungal infections in humans: the silent crisis. Microbial Cell 7:143. 10.15698/mic2020.06.718 10.15698/mic2020.06.718 PMC7278517 32548176

[CR16] Koehler P, Stecher M, Cornely OA, Koehler D, Vehreschild MJ, Bohlius J, Wisplinghoff H, Vehreschild JJ (2019) Morbidity and mortality of candidaemia in Europe: an epidemiologic meta-analysis. Clin Microbiol Infect 25:1200–1212. 10.1016/j.cmi.2019.04.024 31039444 10.1016/j.cmi.2019.04.024

[CR17] Lebreton L, Andrady A (2019) Future scenarios of global plastic waste generation and disposal. Palgrave Commun 5(1):1–11. 10.1057/s41599-018-0212-7

[CR18] Lima R, Ribeiro FC, Colombo AL, de Almeida Jr JN (2022) The emerging threat antifungal-resistant *Candida tropicalis* in humans, animals, and environment. Front Fung Biol 3:957021. 10.3389/ffunb.2022.957021 10.3389/ffunb.2022.957021 PMC10512401 37746212

[CR19] Lockhart SR, Messer SA, Pfaller MA, Diekema DJ (2008) Geographic distribution and antifungal susceptibility of the newly described species *Candida orthopsilosis* and *Candida metapsilosis* in comparison to the closely related species *Candida parapsilosis*. J Clin Microbiol 46:2659–2664. 10.1128/jcm.00803-08 18562582 10.1128/JCM.00803-08 PMC2519489

[CR20] Metcalf R, Oliver DM, Moresco V, Quilliam RS (2022) Quantifying the importance of plastic pollution for the dissemination of human pathogens: The challenges of choosing an appropriate ‘control’ material. Sci Tot Environ 810:152292. 10.1016/j.scitotenv.2021.152292 10.1016/j.scitotenv.2021.152292 34896491

[CR21] Metcalf R, Akinbobola A, Woodford L, Quilliam RS (2025) Thermotolerance, virulence, and drug resistance of human pathogenic Candida species colonising plastic pollution in aquatic ecosystems. Env Sci Pollut Res. In Press. 10.1007/s11356-025-36558-2 10.1007/s11356-025-36558-2 PMC12202632 40465123

[CR22] Moresco V, Charatzidou A, Oliver DM, Weidmann M, Matallana-Surget S, Quilliam RS (2022) Binding, recovery, and infectiousness of enveloped and non-enveloped viruses associated with plastic pollution in surface water. Environ Pollut 308:119594. 10.1016/j.envpol.2022.119594 35680062 10.1016/j.envpol.2022.119594

[CR23] Mpakosi A, Siopi M, Demetriou M, Falaina V, Theodoraki M, Meletiadis J (2022) Fungemia due to *Moesziomyces aphidis* (*Pseudozyma aphidis*) in a premature neonate. Challenges in species identification and antifungal susceptibility testing of rare yeasts. J Med Mycol 32(3):101258 10.1016/j.mycmed.2022.101258 35247802

[CR24] Mphasa M, Ormsby MJ, Mwapasa T, Nambala P, Chidziwisano K, Morse T, Feasey N, Quilliam RS (2025) Urban waste piles are reservoirs for human pathogenic bacteria with high levels of multidrug resistance against last resort antibiotics: a comprehensive temporal and geographic field analysis. J Hazard Mater 484:136639. 10.1016/j.jhazmat.2024.136639 39637810 10.1016/j.jhazmat.2024.136639

[CR25] Nadeem SG, Hakim ST, Kazmi SU (2010) Use of CHROMagar *Candida* for the presumptive identification of *Candida* species directly from clinical specimens in resource-limited settings. Libyan J Med 5(1). 10.3402/ljm.v5i0.2144 10.3402/ljm.v5i0.2144 PMC3071170 21483597

[CR26] Okoye CA, Nweze E, Ibe C (2022) Invasive candidiasis in Africa, what is the current picture? Pathog Dis 80(1):ftac012. 10.1093/femspd/ftac012 10.1093/femspd/ftac012 35451463

[CR27] Opulente DA, Langdon QK, Buh KV, Haase MAB, Sylvester K, Moriarty RV, Jarzyna M, Considine SL, Schneider RM, Hittinger CT (2019) Pathogenic budding yeasts isolated outside of clinical settings. FEMS Yeast Res 19(3):foz032. 10.1093/femsyr/foz032 10.1093/femsyr/foz032 PMC6702360 31076749

[CR28] Ormsby MJ, Akinbobola A, Quilliam RS (2023) Plastic pollution and fungal, protozoan, and helminth pathogens - A neglected environmental and public health issue? Sci Total Environ 882:63093. 10.1016/j.scitotenv.2023.163093 10.1016/j.scitotenv.2023.163093 36996975

[CR29] Ormsby MJ, Woodford L, Quilliam RS (2024) Can plastic pollution drive the emergence and dissemination of novel zoonotic diseases? Environ Res 246:118172. 10.1016/j.envres.2024.118172 38220083 10.1016/j.envres.2024.118172

[CR30] Paredes K, Sutton DA, Cano J, Fothergill AW, Lawhon SD, Zhang S, Watkins JP, Guarro J (2012) Molecular identification and antifungal susceptibility testing of clinical isolates of the *Candida rugosa* species complex and proposal of the new species *Candida neorugosa*. J Clin Microbiol 50(7):2397–2403 22553236 10.1128/JCM.00688-12 PMC3405575

[CR31] Pathadka S, Yan VKC, Neoh CF, Al-Badriyeh D, Kong DCM, Slavin MA, Cowling BJ, Hung IFN, Wong ICK, Chan EW (2022) Global consumption trend of antifungal agents in humans from 2008 to 2018: data from 65 middle-and high-income countries. Drugs 82:1193–1205. 10.1007/s40265-022-01751-x 35960433 10.1007/s40265-022-01751-x PMC9402496

[CR32] Pfaller MA, Diekema DJ, Gibbs DL, Newell VA, Ellis D, Tullio V, Rodloff A, Fu W, Ling TA (2010) Results from the ARTEMIS DISK Global Antifungal Surveillance Study, 1997 to 2007: a 10.5-year analysis of susceptibilities of *Candida* species to fluconazole and voriconazole as determined by CLSI standardized disk diffusion. J Clin Microbiol 48(4):1366–1377. 10.1128/jcm.02117-09 10.1128/JCM.02117-09 PMC2849609 20164282

[CR33] Sharma M, Chakrabarti A (2023) Candidiasis and other emerging yeasts. Curr Fung Infect Rep 17:15–24. 10.1007/s12281-023-00455-3 10.1007/s12281-023-00455-3 PMC9886541 36741271

[CR34] Silva SE, Silva LS, Eufrasio LG, Cruz GS, Lucini F, Vechi HT, do Monte Alves M, Ribeiro LRF, de Souza KL, Moreira JA, de Souza JG (2025) *Kodamaea ohmeri*: an emergent yeast from a one health perspective. Curr Res Microbial Sci 100359. 10.1016/j.crmicr.2025.100359 10.1016/j.crmicr.2025.100359 PMC11926683 40123593

[CR35] Siscar-Lewin S, Hube B, Brunke S (2022) Emergence and evolution of virulence in human pathogenic fungi. Trends Microbiol 30:693–704. 10.1016/j.tim.2021.12.013 35058122 10.1016/j.tim.2021.12.013

[CR36] Trost A, Graf B, Eucker J, Sezer O, Possinger K, Göbel UB, Adam T (2004) Identification of clinically relevant yeasts by PCR/RFLP. J Microbiol Meth 56(2):201–211. 10.1016/j.mimet.2003.10.007 10.1016/j.mimet.2003.10.007 14744449

[CR37] World Health Organization (2022) WHO fungal priority pathogens list to guide research, development and public health action. https://www.who.int/publications/i/item/9789240060241

[CR38] Yan H, Cordier M, Uehara T (2024) Future projections of global plastic pollution: scenario analyses and policy implications. Sustainability 16:643. 10.3390/su16020643

[CR39] Zettler ER, Mincer TJ, Amaral-Zettler LA (2013) Life in the “plastisphere”: Microbial communities on plastic marine debris. Environ Sci Technol 47(13):7137–7146. 10.1021/es401288x 23745679 10.1021/es401288x

[CR40] Zhou M, Li Y, Xu Y (2021) *Kodamaea ohmeri* as an emerging human pathogen: a review and update. Front Microbiol 12:736582. 10.3389/fmicb.2021.736582 34566940 10.3389/fmicb.2021.736582 PMC8461310

[CR41] Zuza-Alves DL, Silva-Rocha WP, Francisco EC, de Araújo MCB, de Azevedo Melo AS, Chaves GM (2019) Candida tropicalis geographic population structure maintenance and dispersion in the coastal environment may be influenced by the climatic season and anthropogenic action. Microbial Pathog 128:63–68 10.1016/j.micpath.2018.12.018 30550843

